# Isolated Axillary Lymphadenopathy Presenting as Carcinoma of Unknown Primary in a Female Patient: A Diagnostic and Therapeutic Challenge

**DOI:** 10.7759/cureus.85842

**Published:** 2025-06-12

**Authors:** Chandana M, Santhoshkumar Bandegudda, Sunitha S

**Affiliations:** 1 General Surgery, Ramaiah University of Applied Sciences, Bangalore, IND; 2 Surgical Oncology, Ramaiah University of Applied Sciences, Bangalore, IND; 3 Pathology, Ramaiah University of Applied Sciences, Bangalore, IND

**Keywords:** axillary lymph node dissection (alnd), carcinoma of unknown primary (cup), immunohistochemistry (ihc), magnetic resonance imaging (mri), positron emission tomography/computed tomography (pet-ct)

## Abstract

Carcinoma of unknown primary (CUP) is a distinct and uncommon clinical entity characterized by metastatic disease without an identifiable primary tumor, despite thorough investigation. Isolated axillary lymphadenopathy as the sole manifestation is rare and diagnostically challenging, particularly in younger women. The patient was a 47-year-old woman with painless right axillary swelling with no breast or systemic lesions. Fine-needle aspiration cytology (FNAC) suggested metastatic carcinoma. PET-CT showed a hypermetabolic right axillary mass without evidence of disease elsewhere. Excisional biopsy confirmed poorly differentiated carcinoma; immunohistochemistry (IHC) was positive for PanCK, CK7, TRPS1, and PAX8 with a high Ki-67 index (70%). A multidisciplinary decision led to axillary lymph node dissection, which revealed no residual metastatic disease. The follow-up PET-CT was normal. This case highlights the importance of a thorough diagnostic approach. IHC, advanced imaging, and multidisciplinary input are critical for diagnosis and management. CUP localized to lymph nodes may have a more favorable prognosis.

## Introduction

Carcinoma of unknown primary (CUP) represents 3% to 5% of all malignancies and is defined by the presence of metastatic disease without an identifiable primary site after comprehensive diagnostic evaluation [1, 2]. The most common sites of metastases are the lymph nodes, lungs, liver, and bones. The diagnosis is often delayed or missed due to the occult nature of the primary tumor. Despite extensive work-up, the primary tumor remains unidentified in up to 70% of cases, even at autopsy [1]. The median age at diagnosis ranges from 60 to 66 years, and the prognosis is typically poor. A meticulous clinical examination, with special attention to the respiratory and abdominal systems, is essential. In women, axillary lymphadenopathy may raise suspicion of an occult breast carcinoma, though other primary tumors must be considered.

## Case presentation

A 47-year-old woman with type 2 diabetes mellitus and no family history of malignancy presented with a 15-day history of painless, progressively enlarging swelling in the right axilla. Clinical examination revealed a 4 x 5 cm solitary, firm, ill-defined mass in the right axilla. No palpable masses were noted in either breast or the contralateral axilla.

**Figure 1:**
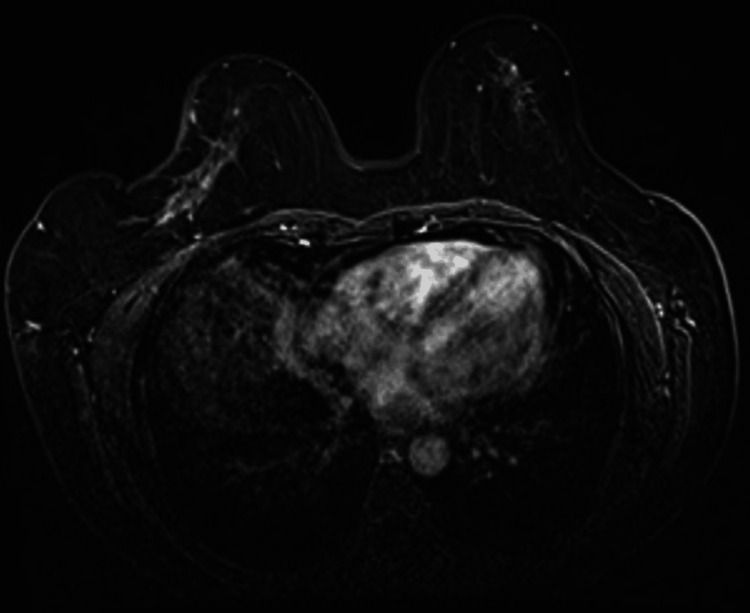
Magnetic resonance mammography (contrast, T1-weighted fat suppressed) showing no lesions in the bilateral breast.

An initial bilateral sonomammography showed an enlarged hyperechoic right axillary lymph node with loss of fatty hilum. Fine-needle aspiration cytology (FNAC) indicated metastatic carcinoma. She was referred to a tertiary care center for further evaluation. Bilateral mammography and breast MRI were unremarkable, as shown in Figure 1.

**Figure 2:**
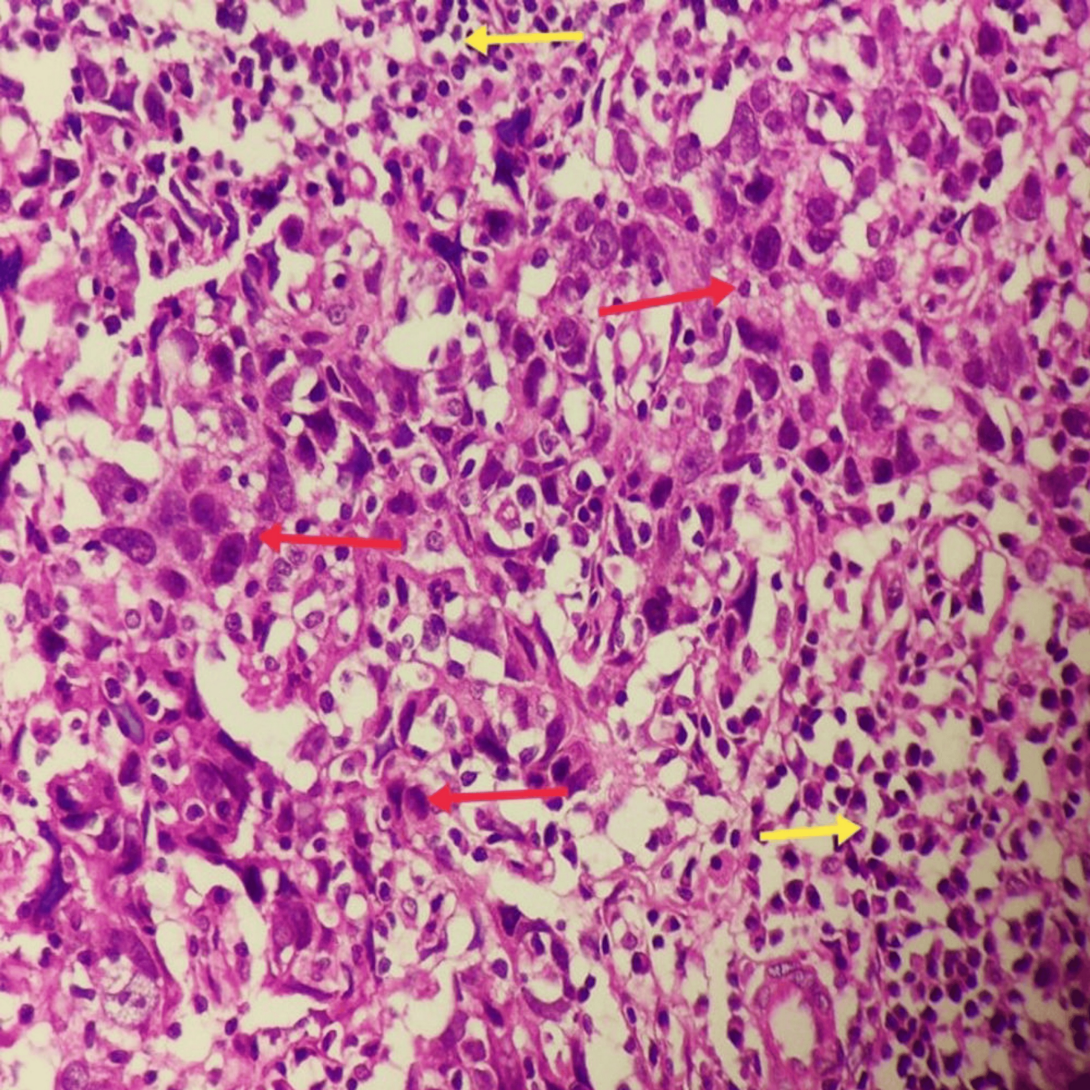
Nests and sheets of poorly differentiated tumor cells (red arrow) are admixed with lymphoid cells (yellow arrow) in the lymph node (400x magnification)

Whole-body PET-CT revealed a hypermetabolic lesion confined to the right axilla with no other abnormalities. Excisional biopsy of the lymph node revealed metastatic poorly differentiated carcinoma depicted by red arrows in Figure 2.

**Figure 3:**
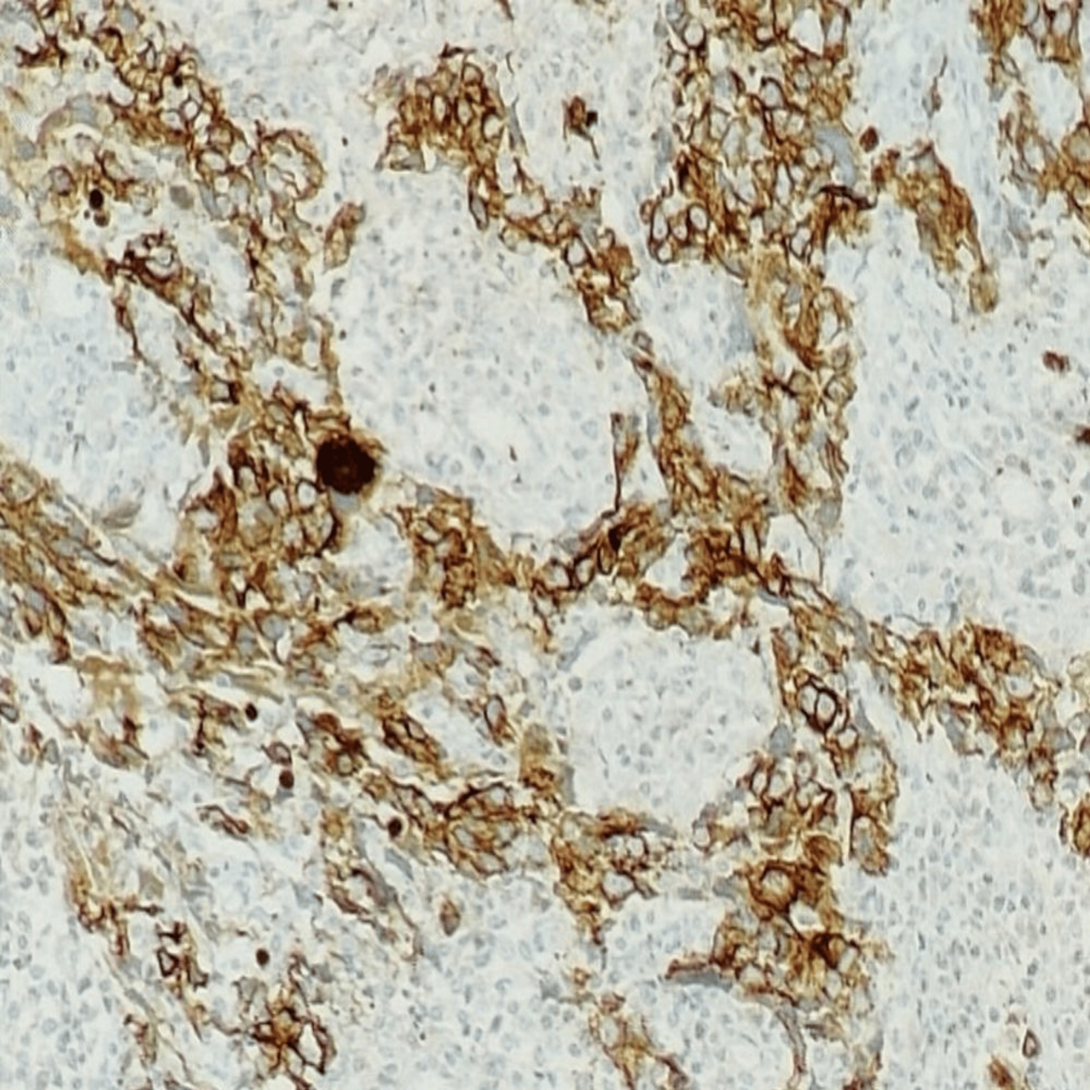
PanCK: Tumor cells are positive and show cytoplasmic staining (IHC, 100x). IHC: immunohistochemistry

**Figure 4:**
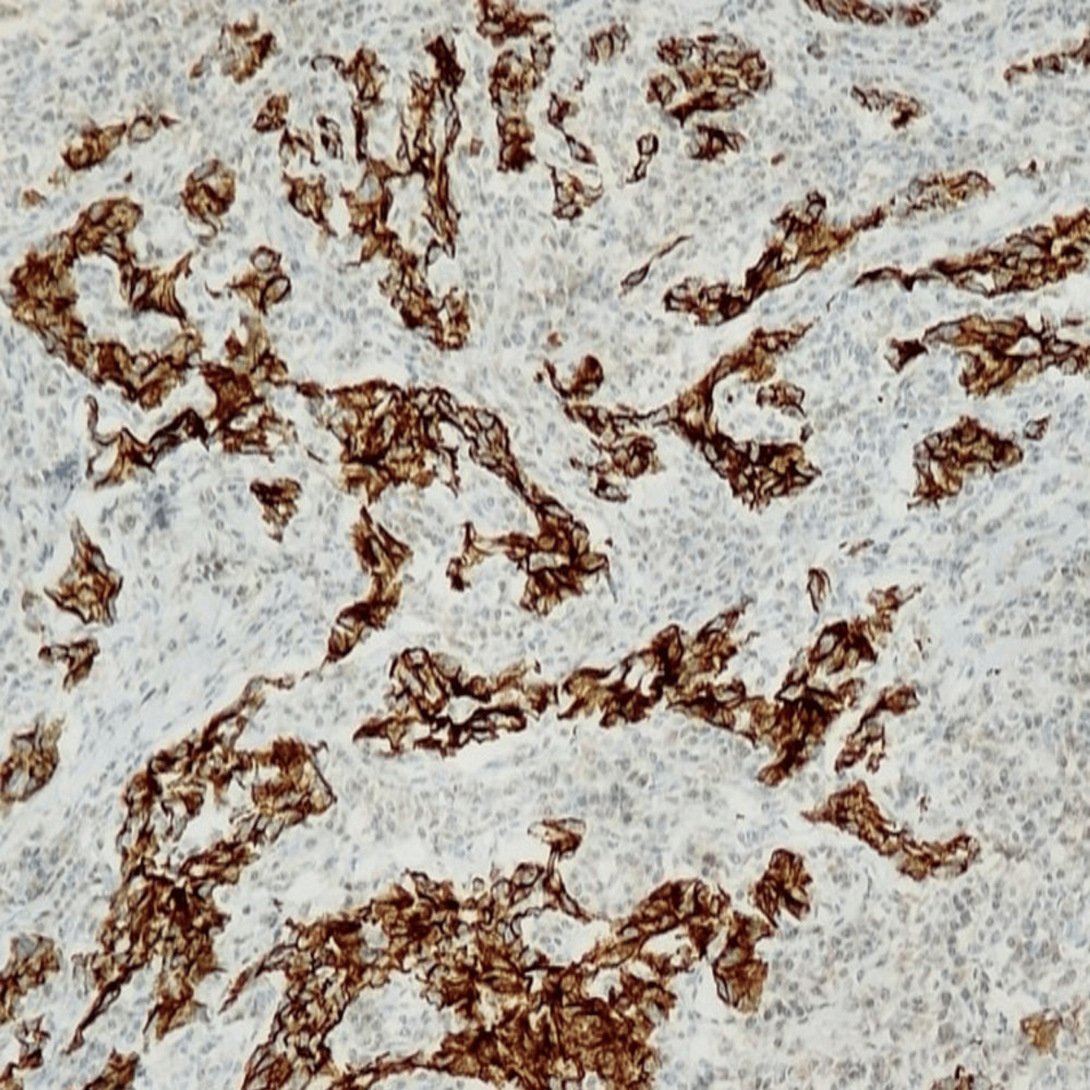
CK 7: Tumor cells are positive and show cytoplasmic staining (IHC, 100x). IHC: immunohistochemistry

**Figure 5:**
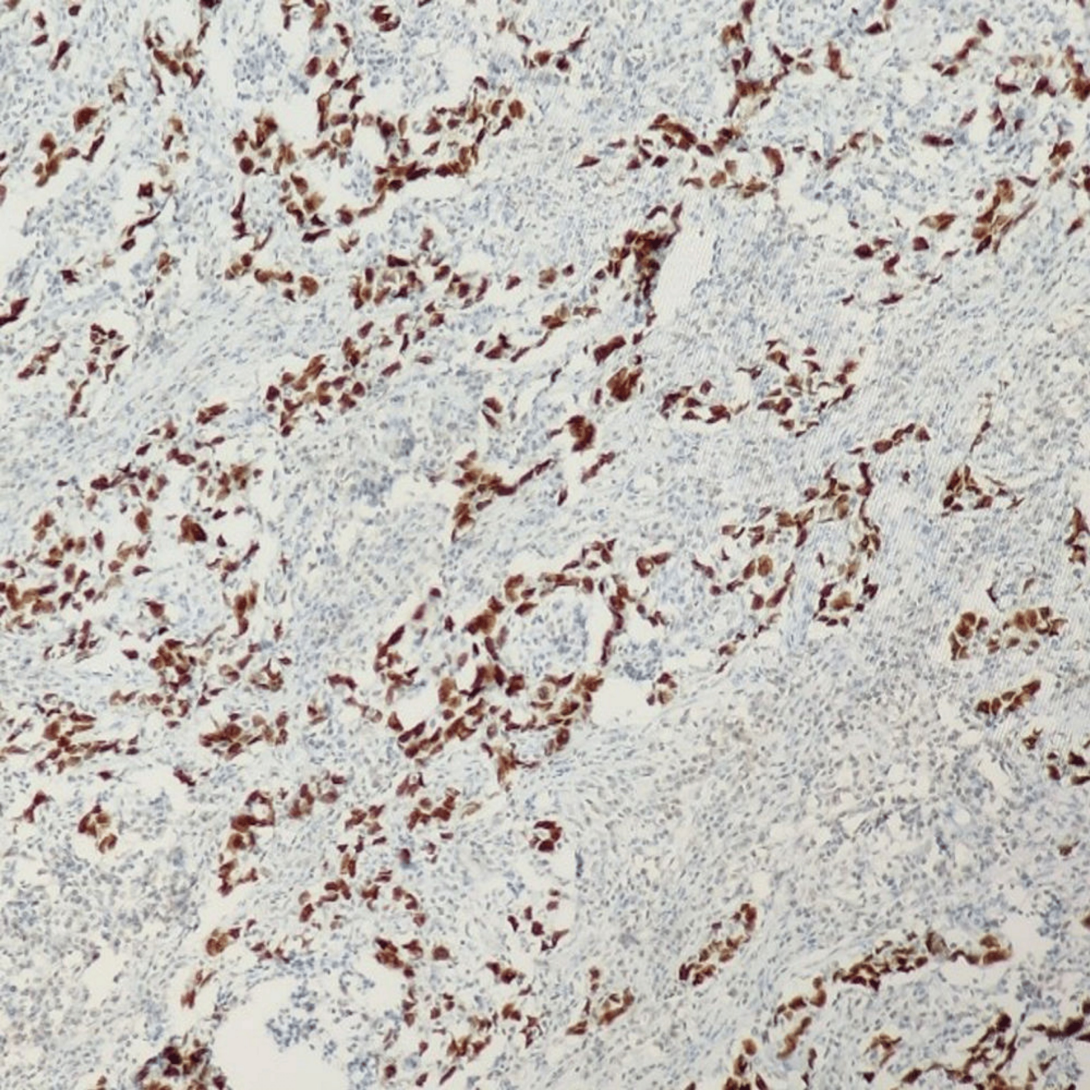
TRPS 1: Tumor cells are positive and show nuclear staining (IHC, 100x). IHC: immunohistochemistry

Immunohistochemistry (IHC) showed positivity for PanCK (Figure 3), CK7 (Figure 4), TRPS1 (Figure 5), and PAX8, and a high proliferative index (Ki-67:70%).

**Figure 6:**
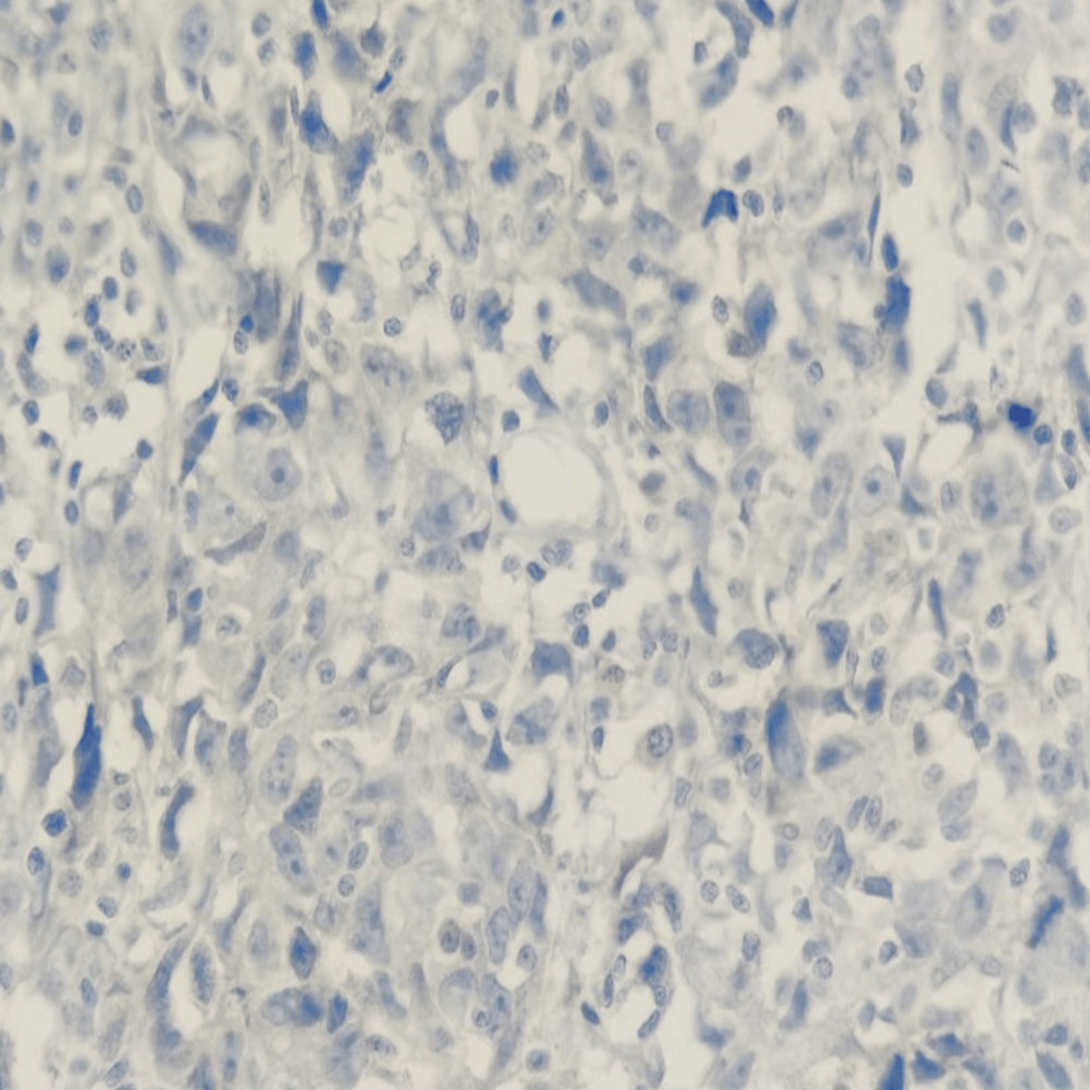
GATA 3: Tumor cells are negative (IHC, 400x). IHC: immunohistochemistry

**Figure 7:**
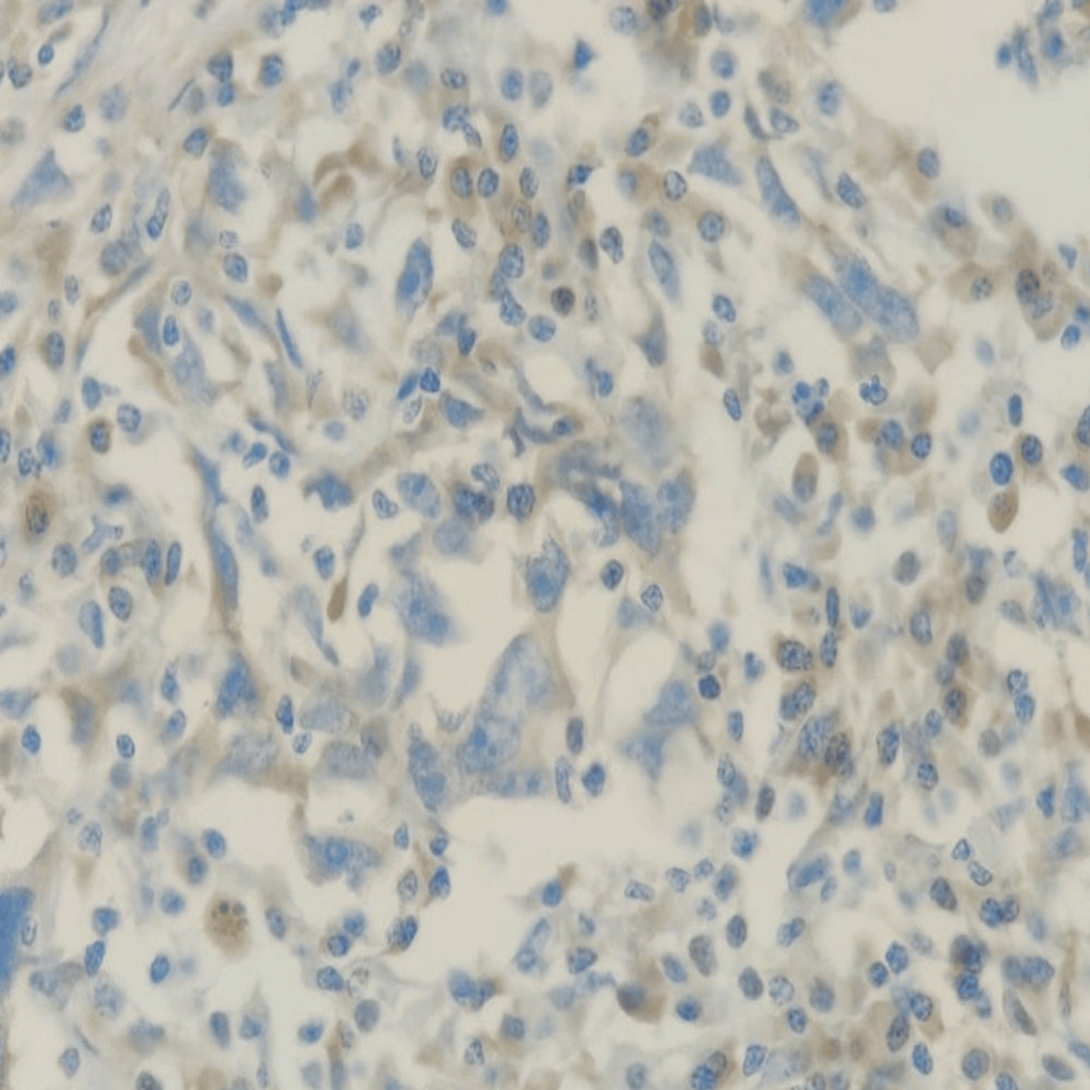
ER: Tumor cells are negative (IHC, 400x). IHC: immunohistochemistry

**Figure 8:**
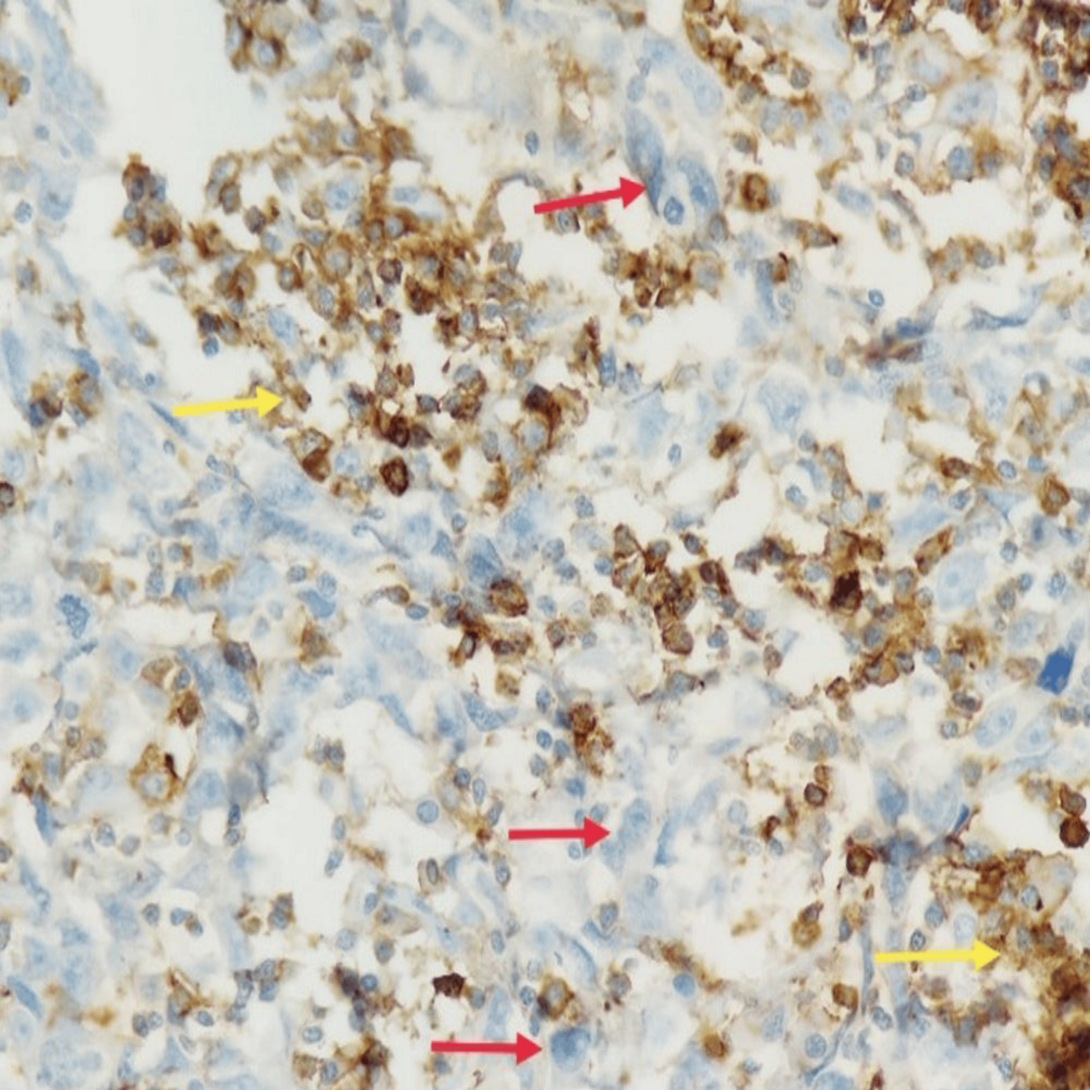
CD45: Tumor cells (red arrow) are negative, and lymphoid cells (yellow arrow) are positive (IHC, 400x). IHC: immunohistochemistry

**Figure 9:**
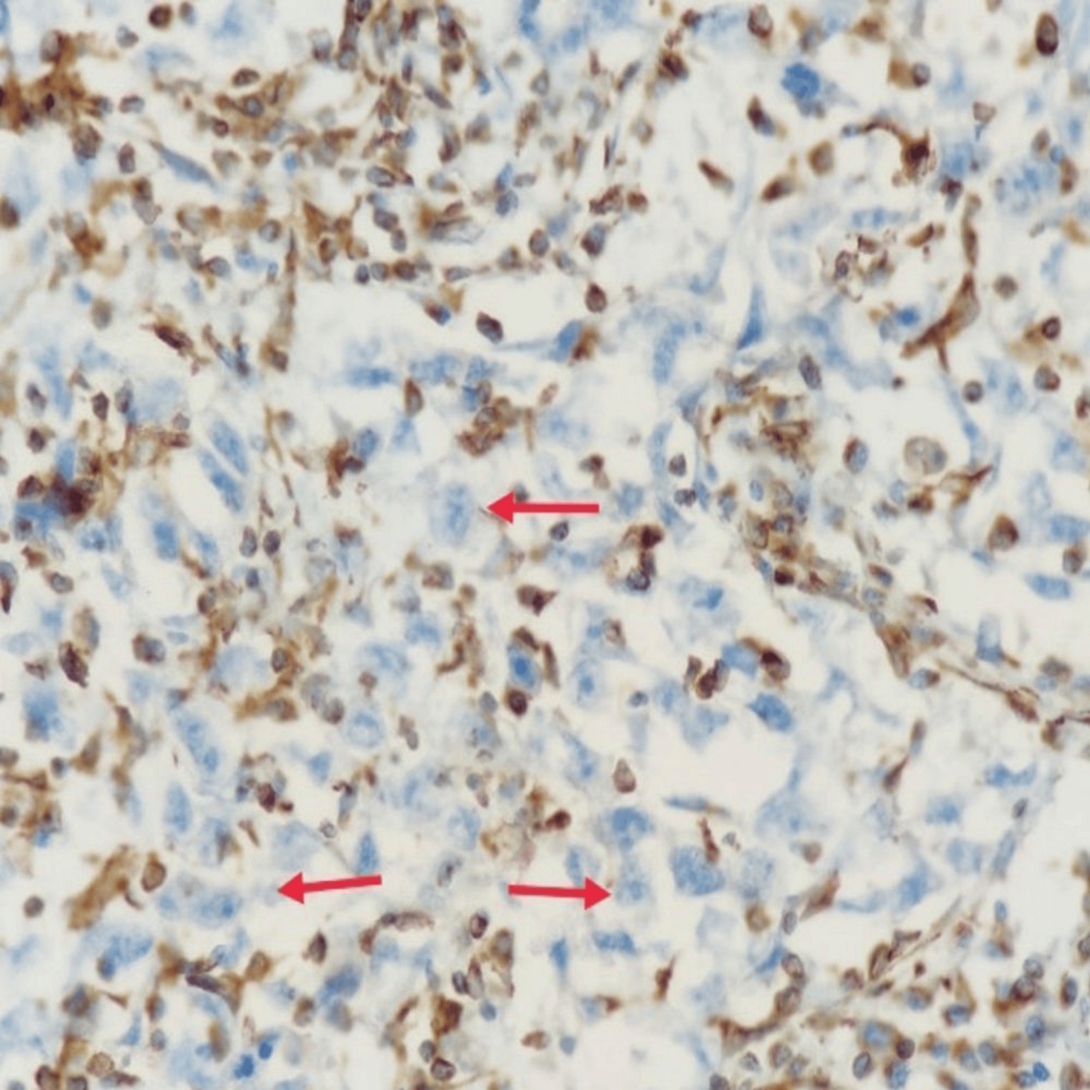
Vimentin: Tumor cells (red arrow) are negative (IHC, 400x). IHC: immunohistochemistry

**Figure 10:**
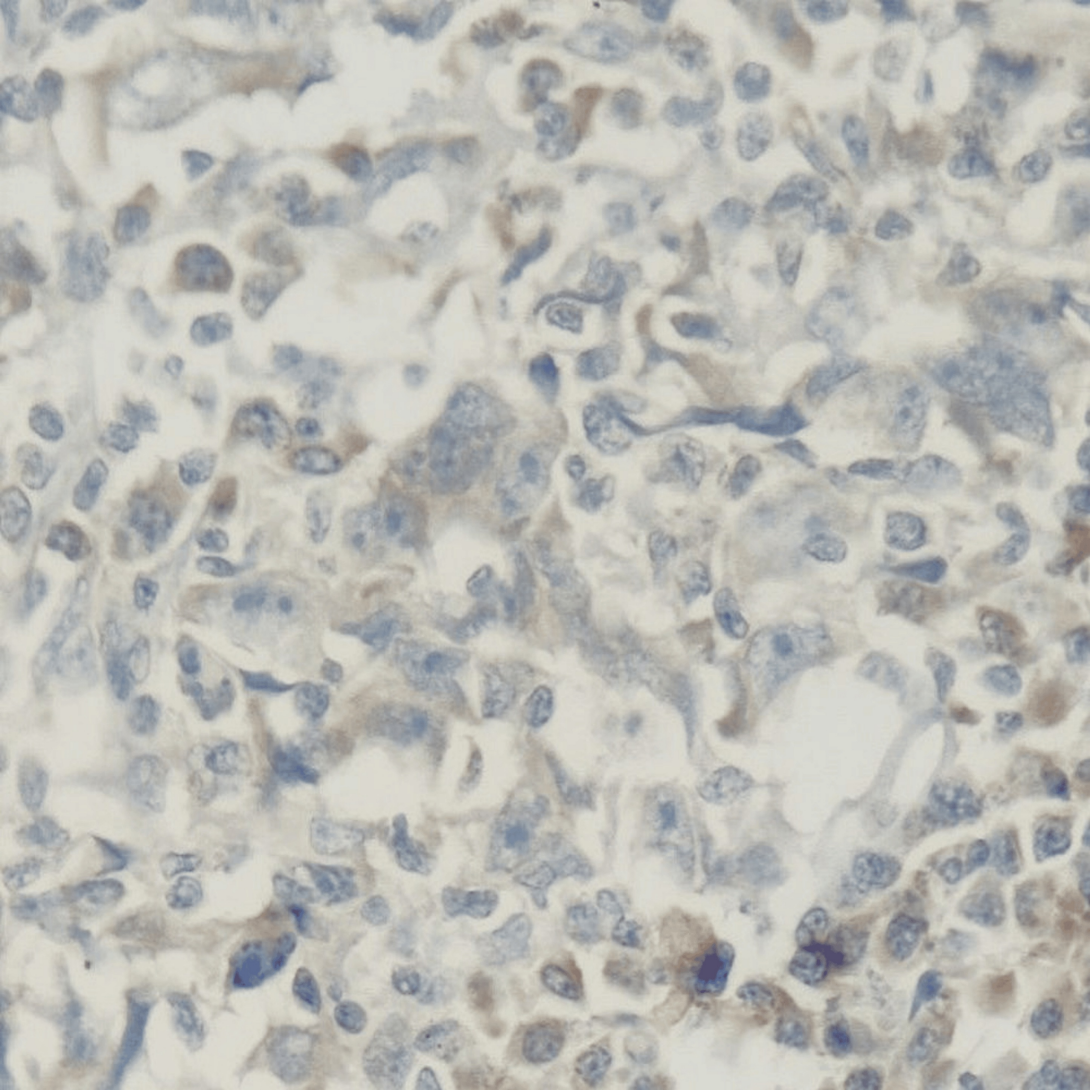
TTF1: Tumor cells are negative (IHC, 400x). IHC: immunohistochemistry

The tumor was negative for markers including CK20, CDX2, Napsin-A, GATA3 (Figure 6), GCDFP-15, mammaglobin, WT1, ER (Figure 7), CD45 (Figure 8), synaptophysin, INSM1, P40, thyroglobulin, CD10, vimentin (Figure 9), and TTF-1 (Figure 10), which ruled out likely origins such as breast, lung, gastrointestinal, and thyroid.

Following multidisciplinary tumor board discussion, the patient underwent a right axillary lymph node dissection (levels I, II, and III). A total of 32 lymph nodes were excised, all free of tumor. She was placed on close follow-up. A repeat PET-CT scan at three months showed no evidence of recurrence or metastasis.

## Discussion

Isolated axillary lymphadenopathy in women is most commonly associated with breast carcinoma. However, in the absence of radiological or clinical breast findings, other primaries must be considered. Common sources of axillary lymph node metastases include malignancies of the breast, thyroid, lung, gastrointestinal tract, ovary, and uterus.

Diagnostic evaluation typically includes ultrasound, mammography, MRI of the breast, CT of the chest, abdomen, and pelvis, and PET-CT [3]. IHC is vital for identifying the likely tissue of origin.

Breast MRI, in particular, has demonstrated superior sensitivity in detecting occult lesions and is endorsed by international guidelines [2].

CUP is hypothesized to follow a distinct “type 2” progression pattern, where metastasis occurs without the development of a clinically detectable primary tumor [1]. Risk factors include smoking, diabetes, autoimmune conditions, obesity, low socioeconomic status, and Black ethnicity [4].

A study by Tang et al. at the West China Hospital analyzed 58 CUP cases and reported 12.07% as poorly differentiated carcinoma and 43 (10%) as invasive carcinoma of no special type. Breast primaries were eventually identified in six patients, two only after prolonged follow-up [5].

Patients with CUP confined to lymph nodes generally have a better prognosis compared to those with extranodal spread [4]. Management often includes surgical excision, especially when nodal involvement is localized and the primary remains unknown.

## Conclusions

CUP presenting as isolated axillary lymphadenopathy in a patient is rare and poses diagnostic challenges. A comprehensive diagnostic approach using advanced imaging and IHC is essential. Surgical management and regular follow-up are key, especially when the disease is confined to lymph nodes. This case emphasizes the potential for favorable outcomes in such presentations of CUP.
